# Comparison between flowable and flowable bulk-fill composites in the manufacture of attachments for orthodontic aligners: a split-mouth randomized clinical trial

**DOI:** 10.1007/s00784-026-07006-3

**Published:** 2026-07-20

**Authors:** Tiago Fialho, Christian Zamberlan Angheben, Eduardo Terumi Blatt Ohira, Paula Cotrin, Daniela Garib, Eduardo Prado, Karina Maria Salvatore Freitas, Marcos Roberto Freitas

**Affiliations:** 1 Department of Orthodontics, Bauru Dental School, São Paulo University, Alameda Octávio Pinheiro Brisolla 9-75, Bauru, São Paulo 17012-901 Brazil; 2 Gaúcho Institute of Postgraduate Studies in Dentistry, Caxias do Sul, Rio Grande do Sul Brazil; 3 UNISOCIESC, Jaraguá do Sul, Santa Catarina Brazil; 4 Ingá University Center, Uningá. Maringá, Paraná Brazil; 5 Prado Institute São Paulo, Moema, São Paulo Brazil

**Keywords:** Orthodontics, Orthodontic appliances, removable, Tooth movement techniques, Malocclusion

## Abstract

**Introduction:**

Orthodontic aligner attachments are crucial for enhancing dental movements that aligners alone cannot achieve. With recent advancements in design and materials, treatment precision and efficacy are improving. This study aimed to compare conventional and bulk-fill flowable composites in fabricating orthodontic attachments to determine their loss rate and clinical outcomes.

**Methods:**

This split-mouth randomized clinical trial enrolled 50 participants (21 men and 29 women; mean age 37.86 ± 6.90 years), who were allocated to two treatment sequences (25 participants per group). In Group 1, the right side received 3 M Filtek Supreme Flowable and the left side received 3 M Filtek Bulk Fill Flowable; in Group 2, the allocation was reversed. This design allowed an intra-individual comparison between materials.

**Results:**

After 140 days of follow-up, 150 of 800 attachments had been lost and rebonded, corresponding to an overall failure rate of 18.75%. No statistically significant differences were found between materials when evaluating individual teeth, contralateral teeth, or time to failure. Cox regression showed no significant difference between groups (HR = 0.84; 95% CI, 0.60–1.16; *p* = 0.293).

**Conclusion:**

Flowable and flowable bulk-fill composites showed similar clinical performance and attachment survival.

**Registration:**

This clinical trial was registered under UTN U1111-1303-5576 at the Brazilian Registry of Clinical Trials (ReBEC).

## Introduction

Attachments are indispensable components in orthodontic aligner therapy, as they enhance aligner retention and facilitate tooth movements that aligners alone cannot efficiently achieve. These movements include rotation of round-shaped teeth, posterior tooth movement, intrusion, and extrusion [1]. Attachments are fabricated based on virtual models generated through specialized software, with each design tailored to fulfill specific biomechanical functions [2].

Since their introduction, orthodontic aligners have undergone significant technological evolution aimed at improving both efficiency and precision. Among these advancements, improvements in attachment design and materials are particularly notable. Nowadays, Invisalign aligners (Align Technology, Inc., Santa Clara, California) employ simple-shaped attachments during the EX30 material era. However, the introduction of the SmartTrack material in 2016 represented a breakthrough, allowing for more complex attachment geometries and expanding the range of achievable movements [3]. These developments have enabled clinicians to manage complex malocclusions, such as pronounced rotations, challenging extrusions, and multi-plane movements, thereby positioning aligners as a viable alternative to fixed appliances in a broader spectrum of cases.

Attachments are applied chairside using templates provided by the aligner manufacturer. The process involves the use of composite resins, with the selection of material varying based on the clinician’s preference, treatment objectives, and case-specific demands. Flowable composites have gained popularity due to their ease of application into the template molds, which reduces the occurrence of voids and simplifies the bonding process [2, 4–6]. Despite these advantages, both conventional and bulk-fill composites remain in widespread use, especially due to their higher filler content, which may offer enhanced mechanical performance.

Multiple factors influence the longevity and effectiveness of attachment. The thickness of the application tray, for example, affects precision and bond strength, and thicker trays offer greater anatomical fidelity. Still, they are associated with higher detachment rates [2]. Patient-related variables such as oral hygiene, diet, and parafunctional habits also play significant roles in attachment survival [6]. Furthermore, appropriate attachment design and positioning, grounded in biomechanical principles, are essential for effective force application and minimizing undesired movements.

This study seeks to contribute to the growing body of evidence regarding attachment fabrication by comparing conventional flowable and bulk-fill flowable composites. By evaluating their detachment rates and clinical outcomes, this investigation aims to determine whether one material offers superior performance and is better suited to the demands of contemporary aligner therapy.

### Specific objectives and hypotheses

This study aimed to evaluate the effectiveness of two different types of flowable composites in fabricating orthodontic attachments. Specifically, it aimed to compare the attachment durability of a conventional flowable composite and a bulk-fill flowable composite.

## Materials and methods

### Trial design

This study is a single-center split-mouth randomized clinical trial with a 1:1 allocation ratio. The Research Ethics Committee approved this prospective study of the Bauru Dental School, São Paulo University _____________ (CAAE 71329423.5.0000.54170_____________), Protocol number 6.577.013___________. This study was also submitted to the Brazilian Registry of Clinical Trials (ReBEC)__________, approved with the identifier UTN U1111-1303-5576_________________. This randomized controlled trial (RCT) followed the Consolidated Standards of Reporting Trials guidelines [7]. 

### Participants, eligibility criteria, and settings

This split-mouth study was conducted in 2024, with patient recruitment carried out at a private orthodontic practice located in Gramado, RS, Brazil__________. Eligibility criteria included male and female patients aged between 18 and 60 years, presenting with Angle Class I malocclusion, complete permanent dentition, good periodontal health, and a maximum Little Irregularity Index (LII) of 2 mm. Exclusion criteria included the presence of incisor agenesis, missing teeth (except third molars), use of bisphosphonate medications, or the need for premolar extraction as part of the orthodontic treatment plan. Patients who met the inclusion criteria were invited to participate, and informed consent was obtained from all participants before their enrollment.

### Interventions

All patients included in the sample were treated with Invisalign orthodontic aligners made of SmartTrack material. During the initial setup, no intermaxillary elastics or dental ramps were used in either group. Furthermore, no interproximal reduction (IPR) was prescribed for the first set of aligners in any participant. All patients underwent the same initial setup, which involved only dental alignment without the use of IPR or any auxiliary mechanics. No transverse expansion or distalization was programmed; thus, the expected dental movements were restricted to proclination. Participants were informed that any additional treatment needs would be addressed in subsequent setups.

Although the sample was matched according to malocclusion characteristics, individual differences in treatment complexity resulted in some patients requiring more aligners than others. To standardize the analysis, all participants were evaluated at the end of the tenth aligner.

Attachments were bonded according to the templates provided for each treatment. The bonding procedure involved etching the tooth surface with 37% phosphoric acid (Dentsply Sirona), followed by rinsing and air-drying. A bonding agent (3 M Adper Single Bond 2) was applied to the etched area and light-cured using the Emitter D curing light (Schuster). All aligners included a Compliance Indicator, allowing verification of proper usage. Additionally, each patient was provided with a daily log chart to record the times they inserted and removed their aligners. These charts served both as a self-monitoring tool and as a method for clinicians to verify patient adherence.

Two flowable composite materials were used for posterior attachments: 3 M Filtek Supreme Flowable (SF) and 3 M Filtek Bulk Fill Flowable (BF), both from São Paulo, Brazil. Each patient’s ClinCheck setup was customized by the primary investigator (TF) to optimize clinical outcomes [8]. 

This study employed a split-mouth design, in which the right and left sides of each participant were treated with different materials, allowing for intra-individual comparison and minimizing the influence of confounding factors, such as age and gender. A total of 50 participants were enrolled (21 men and 29 women), with a mean age of 37.86 years (± 6.90). Interventions and evaluations were conducted unilaterally on each participant.

Participants were then randomly assigned to two subgroups:

Group 1 (G1): Consisted of 25 participants (9 men, 16 women). The right side of each participant received attachments using 3 M Filtek Supreme Flowable (SF), while the left side was treated with 3 M Filtek Bulk Fill Flowable (BF). Patients changed the first ten aligners every 14 days. The mean initial age in this group was 38.56 years (± 7.57).

Group 2 (G2): Comprised 25 participants (12 men, 13 women). In this group, the right side was treated with 3 M Filtek Bulk Fill Flowable (BF) and the left side with 3 M Filtek Supreme Flowable (SF). As in G1, aligners were changed every 14 days. The mean initial age was 37.15 years (± 6.23).

For treatment monitoring, all patients underwent digital intraoral scans at two time points: pretreatment (T1) and after the tenth aligner (T2). Scans were obtained using the iTero Element 5D intraoral scanner (Align Technology, Inc., Santa Clara, CA, USA). The resulting images were stored in a digital database accessible to the lead researcher.

After the second scan (T2), all digital files were uploaded to the OrthoCAD software (Align Technology, San Jose, CA, USA) for analysis. The following measurements were taken from the maxillary and mandibular arches.

### Outcomes (primary and secondary)

The primary outcome was the attachment survival rate, defined as the percentage of attachments that remained bonded to the teeth throughout the observation period [9]. 

**Fig. 1:**
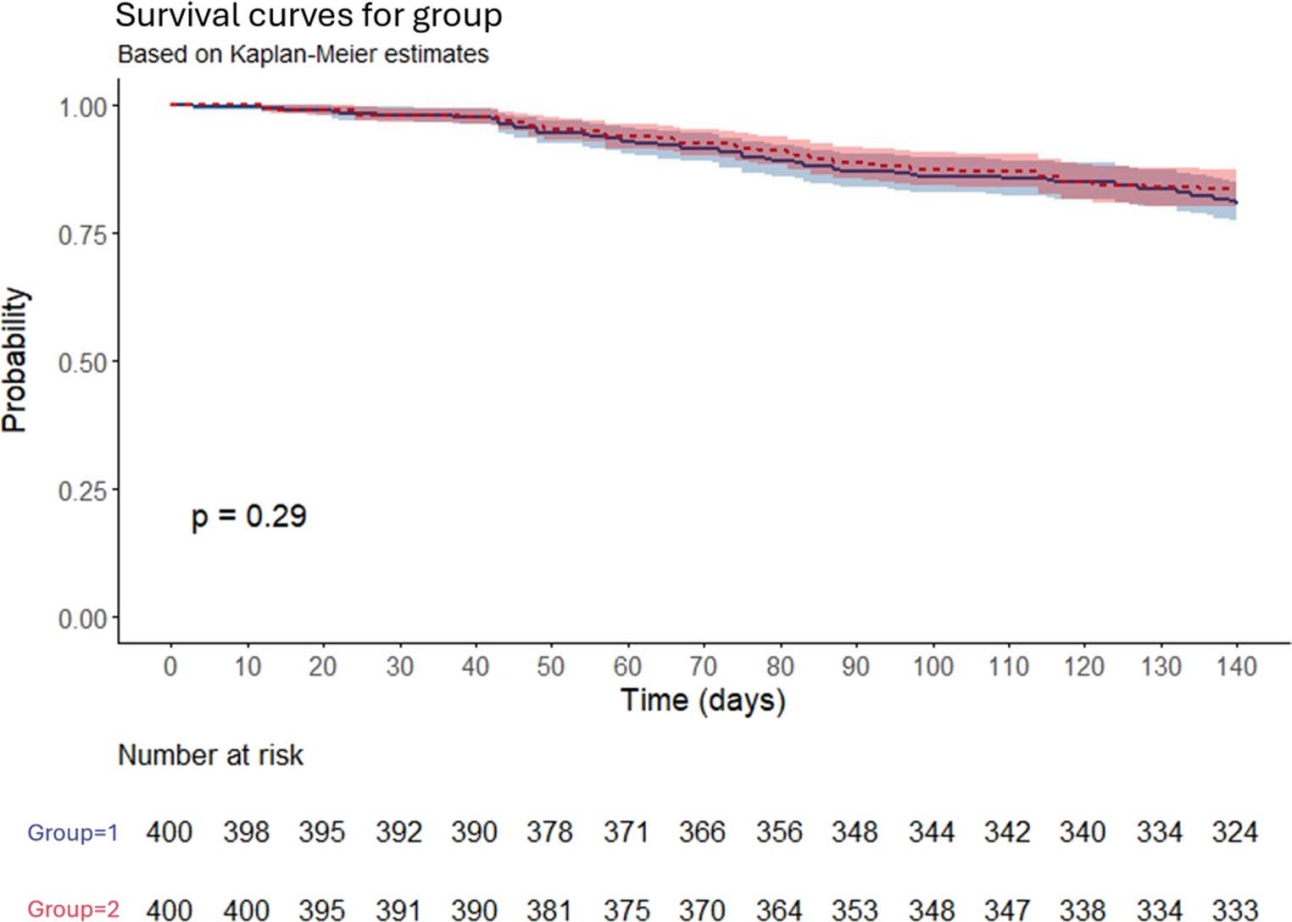
Cox regression based on Kaplan-Meier estimates

The secondary outcome was the time to attachment failure during the 140-day follow-up period, assessed using Cox regression and Kaplan-Meier survival analysis (Fig. 1).

#### Sample size calculation

The sample size calculation was based on a significance level (α) of 5% (0.05), a beta of 80%, and an effect size (f) of 0.30 [5]. Power analysis indicated a requirement of 21 individuals per group, totaling 42 participants.

#### Interim analyses and stopping guidelines

Not applicable.

### Randomization (random number generation, allocation concealment, and implementation)

The blocked randomization process was carried out using the Randomization.com website (http://www.randomization.com). Allocation concealment was ensured through the use of opaque, sealed, and sequentially numbered envelopes, each containing the group assignment according to the randomization sequence [10]. An independent operator, not involved in patient treatment, was responsible for generating the randomization sequence, managing allocation concealment, and implementing the assignments.

The allocation process was initiated after the recruitment of patients who met the inclusion criteria and signed the informed consent form. Before opening each envelope, the patient’s name and allocation date were irreversibly recorded on the external surface. Inside each envelope, a card indicated the group to which the participant had been assigned. To maintain the integrity of the allocation process, all envelopes were opened by tearing (rather than unsealing) and subsequently stored in a secure facility separate from the clinical site.

### Blinding

The operator and patients were aware of the type of treatment performed. However, blinding was performed at the time of measurements. To achieve this, after collecting the final scans (T2), all files were anonymized and analyzed individually by a separate evaluator who was unaware of the patient’s identity, the group to which they belonged, or whether the scan was initial (T1) or final (T2).

### Error study

The error of the method was calculated by remeasuring 30% of the digital files, regardless of whether they were initial or final, with a 30-day time interval. Random error was determined using Dahlberg’s formula. To calculate the systematic error, a paired t-test was used with a significance level of 5% (*P* < 0.05).

### Statistical analyses

The normality of the data was checked with the Shapiro-Wilk test.

The intergroup comparability of sex distribution was calculated with the chi-square test.

Independent t-tests were performed to assess the intergroup comparability of ages at T1.

The intergroup comparisons of all variables studied at the 2 stages evaluated (T1 and T2) were calculated with independent *t-tests*.

The Cox Regression performed the intergroup comparison of the absence of attachments in T2 and the probability of attachment failure at 30-day intervals.

All statistical analyses were performed using the Statistica for Windows software (version 10.0; StatSoft, Tulsa, Okla) at *P* < 0.05.

## Results

### Participants flow

**Fig. 2:**
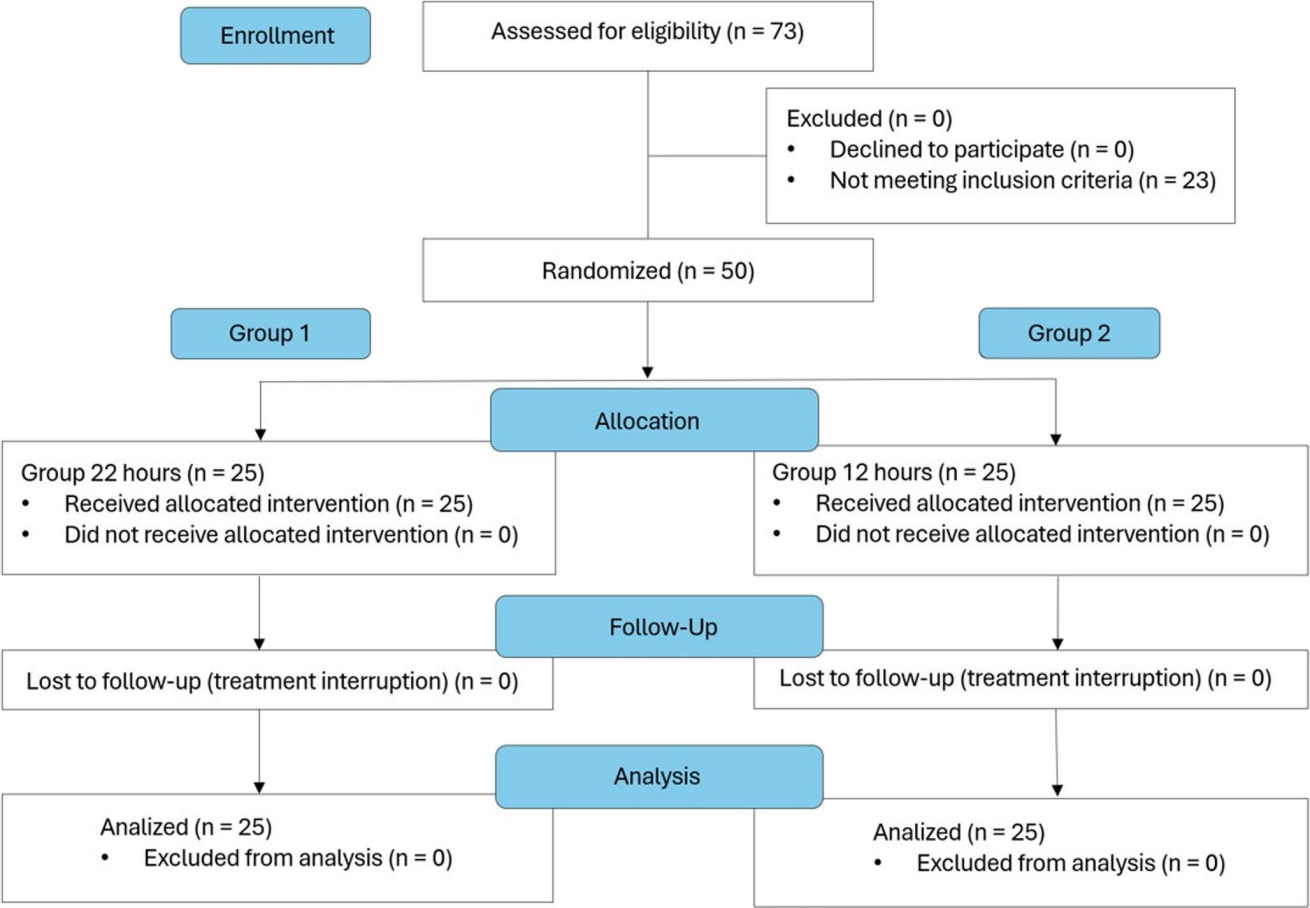
Participant’s flowchart

The flowchart is presented in Fig. 2. During the recruitment phase, 50 patients were assessed for eligibility. The random allocation sequence was generated using a simple random sampling table in Microsoft Excel, with an allocation ratio of 1:1. Allocation concealment was ensured through the use of sequentially numbered, opaque, and sealed envelopes, which were opened immediately before the start of treatment.

Randomization and allocation concealment were performed by the primary supervisor (KF), who was not involved in the clinical treatment of participants. Once a patient agreed to participate and provided informed consent, the primary investigator (TF) retrieved the next envelope in the sequence, opened it, and assigned the patient to one of the two predetermined groups (G1 or G2). All patients assessed for eligibility proceeded to receive the allocated orthodontic treatment.

### Baseline data

Baseline characteristics were similar regarding age and sex distribution (Table 1).

**Table 1:** Results of the intergroup comparison of the absence or presence of attachments in T2 (McNemar’s test)

Tooth	Bulk-fill composite failed		Conventional flowable composite failed		Discordant pairs		
	Yes	No	Yes	No	b	c	*p*-value
-----	--------	------	--------	------	------	------	------
**14**	2	23	1	24	1	0	1.000
**15**	3	22	2	23	1	0	1.000
**16**	7	18	6	19	1	0	1.000
**17**	8	17	8	17	0	0	–
**24**	2	23	2	23	0	0	–
**25**	2	23	2	23	0	0	–
**26**	8	17	9	16	0	1	1.000
**27**	9	16	8	17	1	0	1.000
**34**	1	24	1	24	0	0	–
**35**	2	23	1	24	1	0	1.000
**36**	7	18	7	18	0	0	–
**37**	9	16	8	17	1	0	1.000
**44**	1	24	0	25	1	0	1.000
**45**	3	22	2	23	1	0	1.000
**46**	8	17	4	21	4	0	0.125
**47**	9	16	9	16	0	0	–

McNemar’s test was applied to assess statistical significance in discordant outcomes between bulk-fill and conventional flowable composites. The p-value is reported only when discordant pairs were present (b + c > 0). A dash (–) indicates that the test was not applicable due to complete agreement between treatments

### Numbers analyzed for each outcome, estimation, and precision, subgroup analyses

The random errors varied from 0.09 (maxillary and mandibular Little irregularity index) to 0.32 (maxillary intercanine width). There were no significant systematic errors.

The groups were comparable at T1 regarding age and gender (Table 1).

There was no statistically significant difference between the groups in intergroup comparisons of the absence or presence of attachments in T2 when analyzing all teeth individually (Table 2).

**Table 2:** Cox regression based on Kaplan-Meier estimates

Explanatory	HR (95% CI)	*p*-value
Bulk-fill composite	Reference	-
Conventional composite	0.84 (0.60–1.16)	0.293

There was no statistically significant difference between the groups in intergroup comparisons of the absence or presence of attachments in T2 when analyzing all teeth individually (Table 2) or when comparing each tooth with its contralateral (Table 3).

**Table 3:** Survival summary

					95% confidence interval	
Levels	Time (days)	Number at risk	Number of events	Survival	Lower	Upper
BF group	30 60 90 120 140	392 371 348 340 342	8 21 23 8 18	98.0% 92.8% 87.0% 85.0% 80.5%	96.6% 90.2% 83.8% 81.6% 76.7%	99.4% 95.3% 90.4% 88.6% 84.5%
CF group	30 60 90 120 140	391 375 353 338 333	9 16 21 15 5	97.8% 93.8% 88.5% 84.7% 83.5%	96.3% 91.4% 85.5% 81.3% 79.9%	99.2% 96.2% 91.7% 88.3% 87.2%

There was no statistically significant difference between the groups in the Cox Regression, with *p* = 0.293 (Table 4).

**Table 4:** Cox regression based on Kaplan-Meier estimates

Cox Table - Group			
Explanatory	Levels	All	HR (Univariable)
Group	1	400 (100.0)	-
	2	400 (100.0)	0.84 (0.60–1.16, *p* = 0.293)

HR, Hazard Ratio; CI, 95% Confidence Interval

There was no statistically significant difference between the groups in the Survival Summary based on the Cox Regression (Table 5).

**Table 5:** Survival Summary

					95% confidence interval	
Levels	Time (days)	Number at risk	Number of events	Survival	Lower	Upper
Group 1	30	392	8	98.0%	96.6%	99.4%
Group 1	60	371	21	92.8%	90.2%	95.3%
Group 1	90	348	23	87.0%	83.8%	90.4%
Group 1	120	340	8	85.0%	81.6%	88.6%
Group 1	140	324	18	80.5%	76.7%	84.5%
Group 2	30	391	9	97.8%	96.3%	99.2%
Group 2	60	375	16	93.8%	91.4%	96.2%
Group 2	90	353	21	88.5%	85.4%	91.7%
Group 2	120	338	15	84.7%	81.3%	88.3%
Group 2	140	333	5	83.5%	79.9%	87.2%

### Harms

No significant harm was observed in the patients of this study. Some patients reported slight discomfort in the cheeks and lips while using the first aligners. All aligners were discarded after the patient’s usage.

## Discussion

The composites used in the fabrication of orthodontic attachments play a key role in determining their durability and, consequently, the success or failure of the planned tooth movements [6]. This study aimed to compare the durability of two flowable composites - one with filler and one without - when used to fabricate orthodontic aligner attachments, and to evaluate whether significant differences exist between the materials.

Participants were evaluated over a total period of 140 days (approximately four and a half months), during which they used and replaced ten aligners at 14-day intervals. In total, 800 attachments were assessed, with four attachments placed in each quadrant across 50 patients. By the end of the tenth aligner, 150 attachments had been lost and subsequently rebonded (Table 2), representing a failure rate of 18.75%.

All participants in this study were classified as Angle Class I with mild crowding. Although deep bite has been considered a potential risk factor for increased detachment, it did not appear to influence outcomes in this sample [9]. This may be attributed to the use of virtual models during attachment planning, which take anatomical characteristics into account and position attachments in areas less susceptible to occlusal forces [9]. 

When comparing the two composite groups, the evaluation of individual teeth (Table 2) revealed no statistically significant difference in the presence or absence of attachment at the end of the observation period. Similarly, the analysis of contralateral teeth (Table 3) confirmed that both composites performed comparably. These findings suggest that flowable composites, whether filled or unfilled, offer similar clinical performance in terms of attachment durability.

However, Tables 2 and 3 indicate that molars experienced proportionally higher rates of attachment failure than premolars in both groups. This outcome is likely attributable to mechanical stress during mastication and the repeated insertion and removal of aligners. As molars are typically the first teeth to come into contact during aligner removal, they are subject to increased traction forces. This observation underscores the importance of considering both patient behavior and attachment location in clinical planning.

To evaluate the probability of attachment failure over time, a Cox regression analysis was performed. This survival analysis technique enables the investigation of not only the proportion of failures but also when these failures occurred, considering multiple time intervals throughout treatment (30, 60, 90, 120, and 140 days). In this study, the model was applied to compare the two allocation schemes of composite materials across hemiarches, defined as Group 1 and Group 2.

In Group 1, participants received the conventional flowable composite (SF) on the right side and the bulk-fill flowable composite (BF) on the left side. In contrast, Group 2 received the bulk-fill material (BF) on the right and the conventional flowable composite (SF) on the left. Thus, all participants were exposed to both materials, with the only difference being the side on which they were applied. This design allowed for intra-individual comparison and controlled for potential confounding factors.

Cox regression was used to assess whether the side allocation of materials had any influence on the time to attachment failure. Although the model indicated a hazard ratio of 0.84 for Group 1 compared to Group 2 (Table 4), this difference was not statistically significant (*p* = 0.293). In other words, there was no evidence that the composite’s position (on the right or left side) significantly affected attachment survival.

The survival summary (Table 5) revealed that the number of failures varied between groups at different time points. Group 2 showed slightly higher failure rates at 30 and 120 days, while Group 1 presented more failures at 60, 90, and 140 days. Nevertheless, these variations did not represent a consistent or statistically significant trend favoring either allocation pattern.

When evaluating individual teeth, only the mandibular right second molar showed a notably higher number of failures with the regular composite (SF, *n* = 8) compared to the filled composite (BF, *n* = 4). Despite this observation, the difference was not statistically significant (Table 2). Across the remaining teeth, minor variations in attachment loss were observed between materials, further reinforcing their equivalent clinical behavior.

Based on the results of this study, no statistically significant differences were observed between flowable composites with or without fillers in terms of attachment survival. These findings corroborate those of Lin et al. [6]., who reported no significant difference between flowable and packable composites in a split-mouth design. This evidence provides orthodontists with greater flexibility in clinical decision-making, as unfilled composites are often more cost-effective and more widely available, representing a practical alternative without compromising clinical effectiveness.

To the best of our knowledge, this is the first study to specifically compare flowable composites with and without fillers for the fabrication of orthodontic attachments. While the results indicate no significant differences, further studies with larger sample sizes, longer follow-up periods, and varied clinical conditions are recommended to validate and expand upon these findings. Additionally, investigations into factors such as patient compliance and bonding properties may offer deeper insights into optimizing attachment longevity in aligner-based treatments.

### Limitations

This study examines two types of flowable resins used in the fabrication of attachments for orthodontic aligners. A limitation of this study is the type of sample selected, where all the patients were Class I with mild crowding. Patients with more complex orthodontic treatments, such as posterior crossbite, may present different results from those established here. Further studies are needed to clarify this issue and evaluate these situations.

## Conclusion

- There was no statistically significant difference between flowable and flowable bulk-fill orthodontic attachments.
- Molars experience proportionally higher attachment failure rates compared to premolars.
- Failure rates demonstrated overall similarity by the end of the observation period.
- Regular composites can be a cost-effective and accessible alternative without compromising clinical results, offering flexibility in material choice.

## Data Availability

The data that support the findings of this study are not openly available due to reasons of sensitivity and are available from the corresponding author upon reasonable request.

## References

[1] Karras T, Singh M, Karkazis E, Liu D, Nimeri G, Ahuja B (2021) Efficacy of Invisalign attachments: A retrospective study. Am J Orthod Dentofac Orthop 160(2):250–258. 10.1016/j.ajodo.2020.04.028 10.1016/j.ajodo.2020.04.028 34217574

[2] Jedliński M, Mazur M, Greco M, Belfus J, Grocholewicz K, Janiszewska-Olszowska J (2023) Attachments for the orthodontic aligner treatment-state of the art-A comprehensive systematic review. Int J Environ Res Public Health 20(5):4481. 10.3390/ijerph20054481 36901488 10.3390/ijerph20054481 PMC10001497

[3] Hassanaly T, Rabal-Solans A, Mediero-Pérez MC, Nieto-Sánchez I (2024) A comparison of the upper anterior teeth movements with optimized and conventional attachment. J Clin Exp Dent 16(4):e480–e484. 10.4317/jced.61466 38725817 10.4317/jced.61466 PMC11078502

[4] Iliadi A, Zervou SK, Koletsi D, Schätzle M, Hiskia A, Eliades T, Eliades G (2024) Surface alterations and compound release from aligner attachments in vitro. Eur J Orthod 46(4):cjae026. 10.1093/ejo/cjae026 38884540 10.1093/ejo/cjae026 PMC11181360

[5] Kircelli BH, Kilinc DD, Karaman A, Sadry S, Gonul EY, Gögen H (2023) Comparison of the bond strength of five different composites used in the production of clear aligner attachments. J Stomatol Oral Maxillofac Surg 124(6):101481. 10.1016/j.jormas.2023 37080356 10.1016/j.jormas.2023.101481

[6] Lin S, Huang L, Li J, Wen J, Mei L, Xu H, Zhang L, Li H (2021) Assessment of preparation time and 1-year Invisalign aligner attachment survival using flowable and packable composites. Angle Orthod 91(5):583–589. 10.2319/063020-598.1 33848325 10.2319/063020-598.1 PMC8376162

[7] Moher D, Hopewell S, Schulz KF, Montori V, Gøtzsche PC, Devereaux PJ, Elbourne D, Egger M, Altman DG (2010) CONSORT 2010 explanation and elaboration: updated guidelines for reporting parallel group randomised trials. BMJ 340:c869. 10.1136/bmj.c869 20332511 10.1136/bmj.c869 PMC2844943

[8] Krieger E, Seiferth J, Marinello I, Jung BA, Wriedt S, Jacobs C, Wehrbein H (2012) Invisalign^®^ treatment in the anterior region: were the predicted tooth movements achieved? J Orofac Orthop 73(5):365–376. 10.1007/s00056-012-0097-9 22890691 10.1007/s00056-012-0097-9

[9] Li Q, Yang K (2024) Loss of attachments in patients during orthodontic therapy with clear aligners: A prospective clinical study. Orthod Craniofac Res 27(2):244–250. 10.1111/ocr.12710 37665036 10.1111/ocr.12710

[10] Sayin MO, Akin E, Karaçay S, Bulakbaşi N (2006) Initial effects of the tongue crib on tongue movements during deglutition: a Cine-Magnetic resonance imaging study. Angle Orthod 76(3):400–405. 10.1043/00033219(2006)076[0400:IEOTTC]2.0.CO;2 16637718 10.1043/0003-3219(2006)076[0400:IEOTTC]2.0.CO;2

